# Amphipathic β^2,2^-Amino Acid Derivatives Suppress Infectivity and Disrupt the Intracellular Replication Cycle of *Chlamydia pneumoniae*

**DOI:** 10.1371/journal.pone.0157306

**Published:** 2016-06-09

**Authors:** Leena Hanski, Dominik Ausbacher, Terttu M. Tiirola, Morten B. Strøm, Pia M. Vuorela

**Affiliations:** 1 Pharmaceutical Design and Discovery Research Group, Division of Pharmaceutical Biosciences, Faculty of Pharmacy, University of Helsinki (UHEL), Helsinki, Finland; 2 Natural Products and Medicinal Chemistry Research Group, Department of Pharmacy, Faculty of Health Sciences, UiT – The Arctic University of Norway, Tromsø, Norway; Second University of Naples, ITALY

## Abstract

We demonstrate in the current work that small cationic antimicrobial β^2,2^-amino acid derivatives (Mw < 500 Da) are highly potent against *Chlamydia pneumoniae* at clinical relevant concentrations (< 5 μM, i.e. < 3.4 μg/mL). *C*. *pneumoniae* is an atypical respiratory pathogen associated with frequent treatment failures and persistent infections. This gram-negative bacterium has a biphasic life cycle as infectious elementary bodies and proliferating reticulate bodies, and efficient treatment is challenging because of its long and obligate intracellular replication cycle within specialized inclusion vacuoles. Chlamydicidal effect of the β^2,2^-amino acid derivatives in infected human epithelial cells was confirmed by transmission electron microscopy. Images of infected host cells treated with our lead derivative **A2** revealed affected chlamydial inclusion vacuoles 24 hours post infection. Only remnants of elementary and reticulate bodies were detected at later time points. Neither the EM studies nor resazurin-based cell viability assays showed toxic effects on uninfected host cells or cell organelles after **A2** treatment. Besides the effects on early intracellular inclusion vacuoles, the ability of these β^2,2^-amino acid derivatives to suppress *Chlamydia pneumoniae* infectivity upon treatment of elementary bodies suggested also a direct interaction with bacterial membranes. Synthetic β^2,2^-amino acid derivatives that target *C*. *pneumoniae* represent promising lead molecules for development of antimicrobial agents against this hard-to-treat intracellular pathogen.

## Introduction

*Chlamydia pneumoniae* (alternatively called *Chlamydophila pneumoniae*) is an air-borne respiratory tract pathogen typically causing a long-lasting dry cough with gradual onset [1, 2]. While approximately 70% of acute *C*. *pneumoniae* respiratory tract infections are mild or asymptomatic, a significant fraction of them cause more severe respiratory tract illnesses such as sinusitis, bronchitis and upper airway illnesses [3]. Furthermore, estimated 5–10% of community-acquired pneumonia cases are caused by *C*. *pneumoniae*, and especially the elderly patients often suffer from a severe illness upon encountering this pathogen [4].

*In vitro* susceptibility studies have shown that *C*. *pneumoniae* is sensitive to macrolides, tetracyclines and quinolones, but complete eradication of the infection is challenging. *C*. *pneumoniae* is resistant to sulfa drugs and trimethoprim, and treatment with penicillins can trigger the formation of a persistent state [5]. Treatment of *C*. *pneumoniae* infections is associated with relapsing symptoms and treatment failures even when the first-choice antibiotics are used. Up to 30% of patients with *C*. *pneumoniae*-caused community acquired pneumonia harbor the bacterium in a cultivable form even after the treatment and symptoms have ceased [5, 6]. Upon treatment failures and non-treated infections, the bacterium may convert to a chronic form characterized by morphological, transcriptional and metabolic changes different from the acute infectious phase. This persistent form of infection is ultimately refractory to even prolonged treatment with antibiotic agents [7, 8]. Despite its non-replicative nature, the persistent *C*. *pneumoniae* infection manipulates the host cell metabolism and signaling pathways, and activation of several proinflammatory and proliferative pathways are associated with the infection [9].

*C*. *pneumoniae* represents an atypical respiratory tract pathogen by being a gram-negative bacterium with an obligate intracellular replication cycle. Characteristic for the life cycle of *C*. *pneumoniae* are successive conversions between the extracellular, non-replicative but infectious elementary bodies (EBs), and the intracellular replicating reticulate bodies (RBs). Bacteria in the genus *Chlamydia* resemble other gram-negative bacteria as they have an outer membrane rich in negatively charged lipooligosaccharides (LOS) [10]. Recently, it has also been shown that *Chlamydia* spp. possess a peptidoglycan cell wall [11]. Cysteine-rich proteins are abundant in the proximity of the outer membrane of the *Chlamydia* cell envelope. These form extraordinary intra- and intermolecular disulfide cross-linkages involving proteins such as the major outer membrane protein (MOMP) and other periplasmic cysteine-rich proteins, which are suspected to play a crucial role in the structural rigidity and osmotic stability of the EBs form [12, 13]. Reduction of the disulfide bonds as well as opening of the supramolecular protein complexes occurs upon differentiation of the EBs to RBs shortly after entering the host cell and contributes to the fragile and osmolabile nature of the intracellular RB form [14]. *Chlamydia* spp. have developed means to support their intracellular survival. The formation of an inclusion vacuole, which is detached from the host cell’s endocytic vesicular system, is essential upon the bacterium’s entry into the host cell [15]. The inclusion membrane provides a protective niche by being an additional permeability barrier against cellular defense mechanisms and antimicrobial agents, and offers a surface for anchoring bacterial and host cell proteins favorable for the infection.

The negatively charged lipooligosaccharides embedded in the outer membrane of *C*. *pneumoniae* display a possible and favorable target structure for treatment with cationic antimicrobial peptides (AMPs). AMPs are a structurally diverse class of naturally existing anti-infective agents forming an essential component of the innate immune system of all multicellular organisms [16]. The antimicrobial activity of the majority of these peptides is tightly linked to their ability to electrostatically interact, insert and finally disrupt bacterial membranes. Selective interaction of AMPs with microbial membranes is explained by a higher negatively charged outer surface and lack of cholesterol compared to eukaryotic cell membranes. Similar observations have been made on cancer cells and several AMPs are regarded as promising cancer therapeutics, especially for treatment of resistant tumors [17, 18]. In order to have antimicrobial or anticancer properties, AMPs require in general a certain content and distribution of cationic and lipophilic residues enabling formation of amphipathic peptide conformations, rather than the presence of specific amino acid sequences [19].

The use of AMPs as potential pharmaceutical agents has been investigated during the last three decades. However, an inherent obstacle in using peptides as therapeutic agents is related to their unfavorable pharmacokinetic properties like compromised oral bioavailability, and low metabolic and proteolytic stability. Thus, most AMPs in clinical trials are developed as topical formulations [20]. A strategy to overcome these obstacles is to prepare peptidomimetics, which are molecules that maintain or improve essential peptide functionalities with respect to biological activity. These molecules are less susceptible to enzymatic degradation and have otherwise more favorable pharmacokinetic properties [21]. We have reported the synthesis of a series of peptide mimicking β^2,2^-amino acid derivatives that resemble small AMPs and additionally fulfill the physicochemical criteria set for orally bioavailable drugs [22]. These derivatives show activity against both gram-positive and gram-negative bacteria with minimum inhibitory concentration values between 4–8 μM while being basically nontoxic against human red blood cells. Of importance to the present study is the observation that β^2,2^-amino acid derivatives are able to permeate through artificial cell membranes by passive diffusion, and our hypothesis thereby became that they also could target intracellular residing pathogenic bacteria such as *C*. *pneumoniae* [22]. It has been reported that AMPs are able to effectively kill metabolically inactive extracellular EBs of *C*. *pneumoniae and C*. *trachomatis* [23]. However, bactericidal effects of AMPs on intracellular residing *Chlamydia* spp. is to our knowledge not reported.

**Fig 1:**
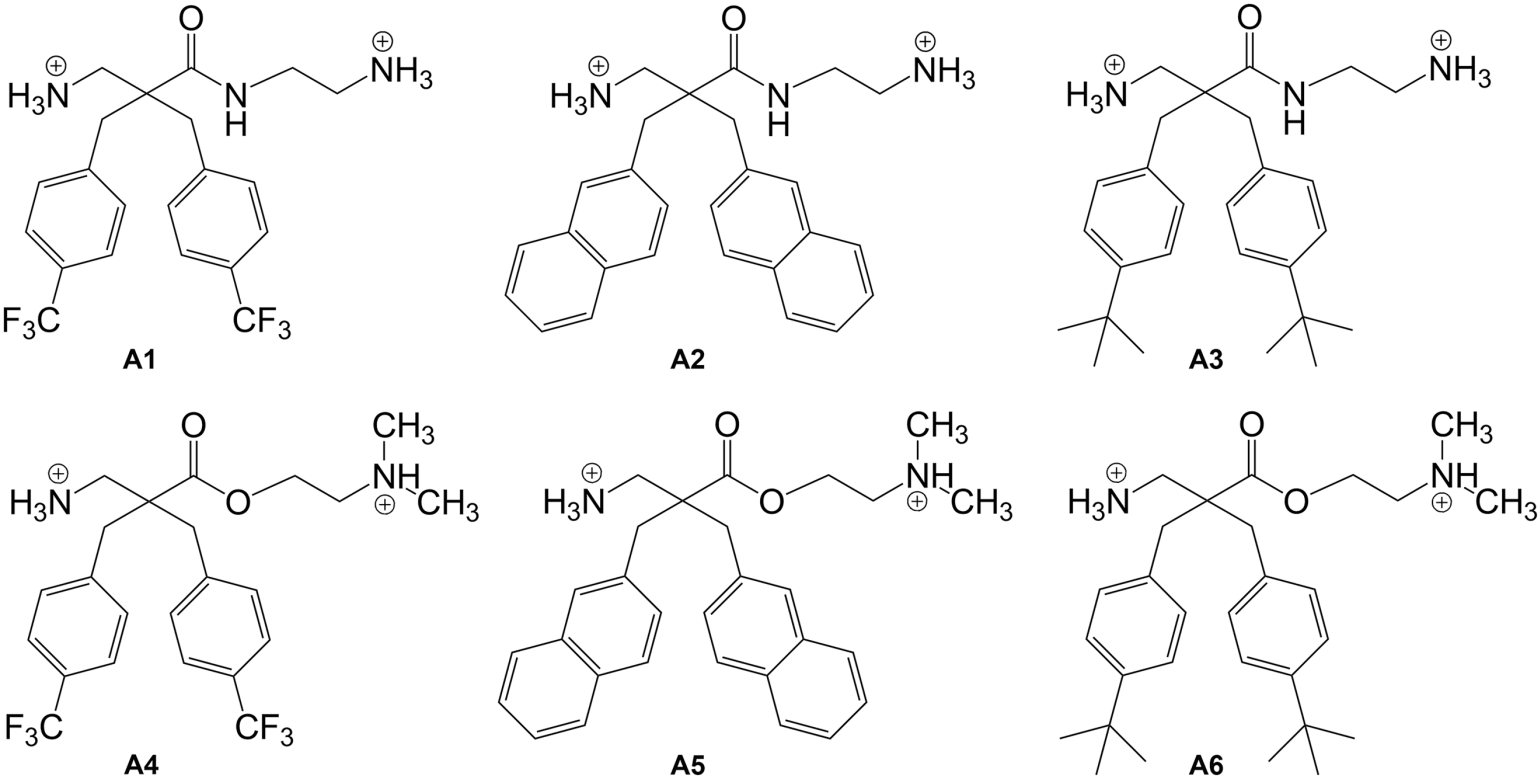
Structures of the investigated β^2,2^-amino acid derivatives A1—A6. All derivatives were isolated as di-trifluoroacetate salts.

In the current work, we demonstrate that derivatives **A1** and **A2**, two out of our six studied β^2,2^-amino acid derivatives (Fig 1), are highly potent and able to inhibit *Chlamydia pneumoniae* infectivity and interfere with *C*. *pneumoniae* inclusions in human epithelial HL cells at concentrations below 5 μM (3.4 μg/mL). We hypothesize that the antichlamydial properties are based on a dual mechanism of action of these derivatives affecting both the EBs and early intracellular inclusions of *C*. *pneumonia*.

## Materials and Methods

### Derivatives A1—A6

The derivatives **A1**—**A6** were synthesized according to previous publications and isolated as di-trifluoroacetate salts [22, 24]. Purity was determined to > 95% with an analytical RP-HPLC C_18_-column and UV detection at 214 nm and 254 nm.

### Cells and bacteria

Human epithelial HL cells [25] were grown in a RPMI1640 medium supplemented with 7.5% fetal bovine serum (FBS), 2 mM *L*-glutamine and 20 μg/ml gentamicin sulfate. A clinical *C*. *pneumoniae* isolate Kajaani 7 (K7), originally isolated from a young Finnish man suffering from pneumonia [26] was obtained from Professor Pekka Saikku (Department of Medicinal Microbiology, Institute of Diagnostics, University of Oulu, Finland) and a cardiovascular *C*. *pneumoniae* isolate CV-6 was obtained from Professor Matthias Maass (Paracelsus Medical University, Salzburg, Austria). The reference strains CWL-029 (VR-1310) and AR-39 (ATCC 53592) were obtained from American Type Culture Collection (ATCC). All strains were propagated in HL cells as previously described [27]. The K7 strain was used in all studies whereas CV-6 and the ATCC reference strains were used to confirm our initial compound screening data.

### Infections

For all experiments applying immunofluorescence, HL cells were seeded in 24-well plates with coverslips at density of 4 x 10^5^ cells/well and incubated overnight before infection. The cells were inoculated with *C*. *pneumoniae* at multiplicity of infection (MOI) 0.2 in the cell growth media supplemented with 1 μg/ml cycloheximide to enhance chlamydial growth [28]. The inoculated cultures were centrifuged at 550 *g* at 4°C for 1 h and incubated at 37°C for 1 h. Inocula were removed, fresh medium was added into wells and the plates were incubated for 70 h. Afterwards the cultures were fixed with methanol and the coverslips were stained with *Pathfinder Chlamydia cell culture confirmation system* (Bio-Rad) based on a genus-specific anti-LPS antibody to visualize the host cells and chlamydial inclusions. Detailed protocols for the infection and staining can be found in [29]. Unless otherwise stated in the text, the derivatives were added into the culture medium at 2 hours post infection (hpi), i.e., at the time when inocula were removed. Rifampicin (Fluka) at a concentration of 12 nM and azithromycin (Sigma) at a concentration 20 nM were used as a positive control in the infections, consistently yielding 95–98% inhibition in *C*. *pneumoniae* inclusion counts. DMSO concentration was adjusted to 0.1% in all experiments.

### Delayed administration assay

HL cells were infected as described above and treated with derivatives at concentrations of 3 μM in order to cause inhibition of infection above 50%. The treatment of the infected cells was delayed by 4, 10 and 22 h and inclusions counts were subsequently performed as described above.

### Infectious progeny assay

To measure the effects of the β^2,2^-amino acid derivatives on production of new infectious progeny by *C*. *pneumoniae*, HL cells were infected with *C*. *pneumoniae* as described above in the presence of the derivatives (at a concentration of 5 μM). At 72 hpi, medium was removed from the wells and 200 μl fresh medium was added. The cells were scraped off and then further lysed by vortexing with glass beads, and the lysates were used to infect fresh HL cell monolayers. Inclusion counts in the second passage of infection were determined with immunofluorescent staining as described above, and control wells fixed and stained after the first infectious cycle were used as internal controls.

### Elementary body infectivity assay

*C*. *pneumoniae* EBs were suspended into the HL cell growth medium supplemented with cycloheximide at 400 000 inclusion forming units (IFU)/ml and exposed to 5 μM of the derivatives **A1**—**A6** for 1 h at 4°C. Immediately afterwards, aliquots of the EBs suspensions were used to infect HL cell monolayers as described above (MOI 0.2), resulting in typically 40–60 inclusions per eye field in nontreated control infections.

### Transmission electron microscopy

HL cells were seeded in 6-well plates at 2 x 10^6^ cells per well and cultured overnight. The cell monolayers were infected with *C*. *pneumoniae* (MOI 1) and treated with 2.5 μM of derivative **A2** by adding it to the culture medium at 2 hpi based on findings in the dose-response assay. Untreated and uninfected HL control cells were incubated concurrently. The cells were pre-fixed at 24, 48 and 72 hpi with Karnovsky's fixative at 4°C, overnight. The samples were further processed for TEM as described in [30].

### Cell viability assays

HL cells were seeded into 96-well plates at density 6 x 10^5^ cells per well and incubated overnight before starting the exposure. The β^2,2^-amino acid derivatives were added at concentrations of 5 μM either in the presence or absence of 1 μg/ml cycloheximide in a final volume of 200 μl and the plates were incubated for 72 h. The resazurin assay was performed as described in Karlsson *et al*. [31].

### Data analysis

All experiments were carried out as four replicates in minimum and the inclusion counts were determined as mean ± SEM of 4 eye fields per coverslip. For statistical analysis GraphPad Prism software v. 5.0 and Student’s *t*-test (p < 0.05) were applied.

## Results

### Inhibition of *C*. *pneumoniae* growth by β^2,2^-amino acid derivatives

**Fig 2:**
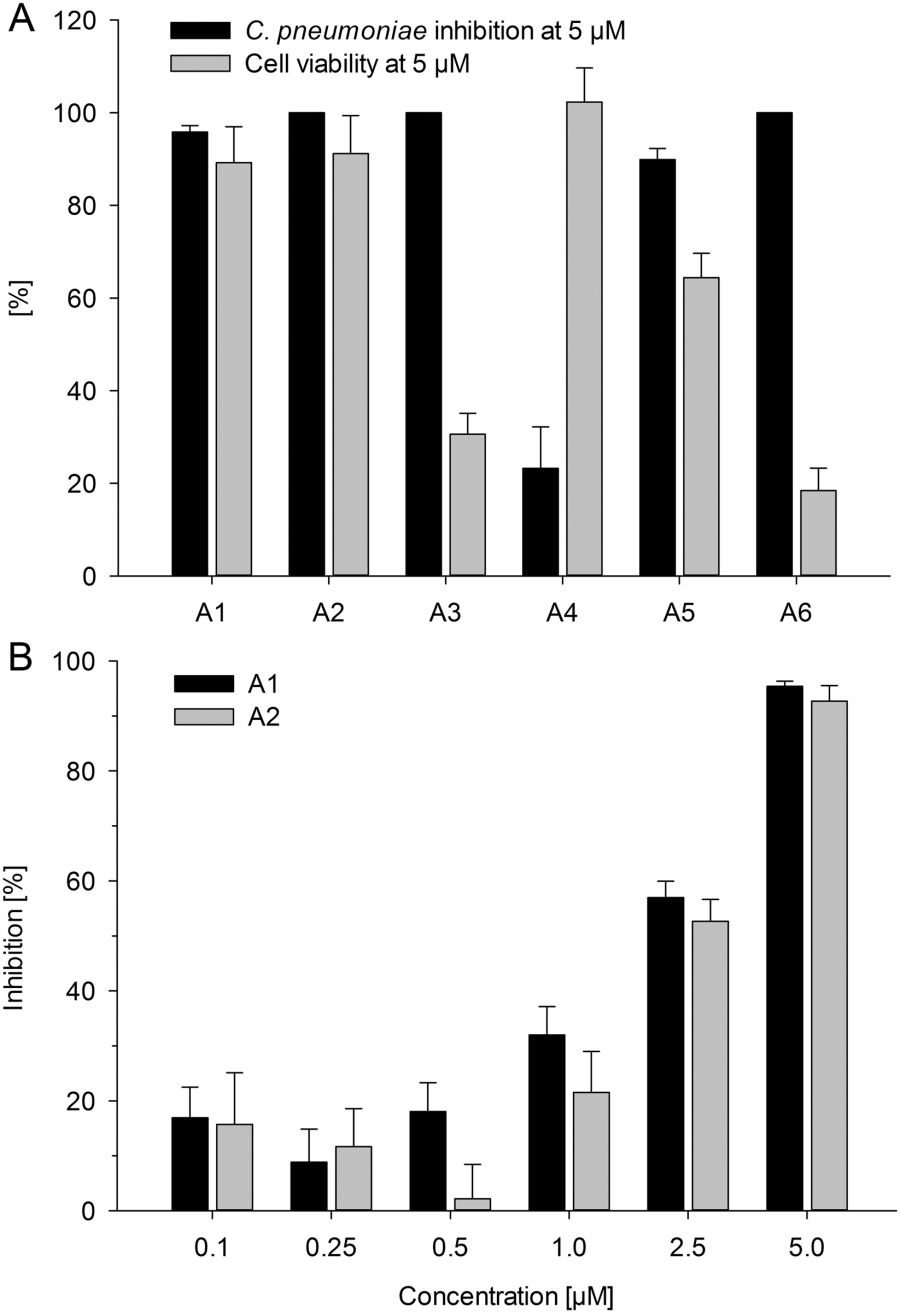
Impact of β^2,2^-amino acid derivatives on *C*. *pneumoniae* inclusion counts and host cell viability. (A) Screening of the β^2,2^-amino acid derivatives **A1**—**A6** for anti-*C*. *pneumoniae* activity and cytotoxicity at concentrations of 5 μM. (B) Dose-response relationship of **A1** and **A2** for decreasing *C*. *pneumoniae* inclusion counts. (Results display the mean ± SEM).

Six β^2,2^-amino acid derivatives, comprising three different lipophilic side-chains and two different cationic *C*-termini (Fig 1) were assayed in a protocol in which human HL epithelial cell monolayers were infected with *C*. *pneumoniae* strain K7. At a concentration of 5 μM (**A1**, 3.4 μg/mL; **A2**, 3.2 μg/mL) the two derivatives, **A1** and **A2**, yielded 95.6% and 100% reduction in inclusion counts with no substantial effect on host HL cell viability (Fig 2A). The derivatives **A3** and **A6** caused full inhibition of *C*. *pneumoniae* infectivity, however, we observed that host HL cells started to detach from the coverslips. The cell viability assay confirmed the rather cytotoxic properties of these two derivatives against HL cells and viability was determined to be 30.6% for **A3** and 18.4% for **A6**. Derivative **A4** showed 23.2% inhibition against *C*. *pneumoniae* and no cytotoxic effects against HL-cells whereas **A5** caused 89.8% inhibition against *C*. *pneumoniae* and a 35.6% reduction of HL cell viability. As shown by the data from dose-response experiments, the anti-chlamydial effect of **A1** and **A2** was dose-dependent and IC_50_ values of 2 μM (**A1**, 1.4 μg/mL; **A2**, 1.3 μg/mL) were obtained for both compounds (Fig 2B). The antichlamydial efficacy of **A1** and **A2** was additionally confirmed using two ATCC reference strains, AR-39 and CWL-029, and a cardiovascular *C*. *pneumoniae* isolate CV-6. These strains showed similar susceptibility to treatment with 5 μM of these derivatives as observed for the K7 strain (data not shown).

### Impact of administration time on *C*. *pneumoniae* inhibition

**Fig 3:**
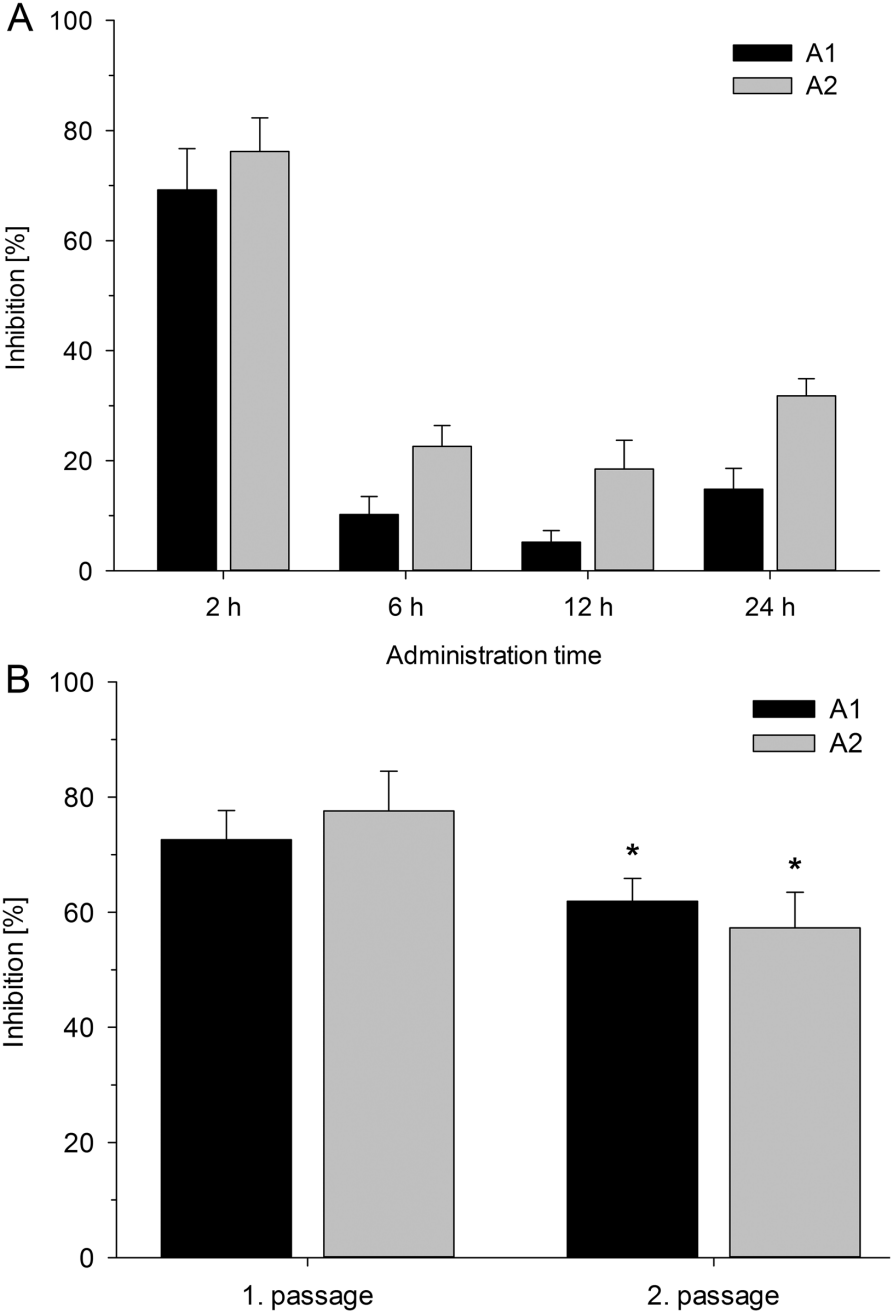
Impact of delayed A1 and A2 administration and inhibition of infectious progeny production by the derivatives. (A) Inhibition of *C*. *pneumoniae* infectivity after delayed administration of 3 μM of derivatives **A1** and **A2.** (B) Inhibition of infectious progeny production by **A1** and **A2** at a concentration of 5 μM. (Results display the mean ± SEM; asterisk indicate significant difference, p < 0.05).

To further analyze the inhibitory properties of the two most promising β^2,2^-amino acid derivatives on *C*. *pneumoniae* growth, **A1** and **A2** were investigated in a delayed administration experiment. The addition of the derivatives into the culture medium of infected cell monolayers was postponed within the *C*. *pneumoniae* 72 h replication cycle. As indicated by the columns presented in Fig 3A, adding 3 μM of **A1** into the culture medium according to the standard procedure (administration at 2 h, i.e. at the time of inocula removal) resulted in approximately 70% inhibition of inclusion vacuole counts, as expected. When **A1** was added at 6 hpi or later, the inhibitory effect was significantly decreased to approximately 10–20% reduction in inclusion vacuole counts. Derivative **A2** followed the same pattern with approximately 80% reduction of inclusion vacuole counts when added at 2 hpi, and showed only inhibiting effects on inclusion vacuole counts in a range between 20–30% when added at any later time points. The size of the remaining inclusion vacuoles did not differ from those in untreated control infections at any time point. Thus, the most pronounced effect caused by **A1** and **A2** on *C*. *pneumoniae* growth was exerted during the early stages of the infectious cycle.

### Impact on infectious progeny production

The ability of **A1** and **A2** to suppress the *C*. *pneumoniae* production of new infectious progeny was studied to gain more insight into the anti-chlamydial effects of these two derivatives. The results presented in Fig 3B show that inclusion vacuole counts detected in the second passage of infection were significantly lower in **A1** and **A2** treated infections than the inclusion vacuole counts of an untreated control infection. Consistent with the data obtained from the administration time experiments, neither **A1** nor **A2** suppressed *C*. *pneumoniae* infectious progeny production when they were administered into infected cell cultures at 6 hpi or later (data not shown).

### Effects on *C*. *pneumoniae* elementary bodies

**Fig 4:**
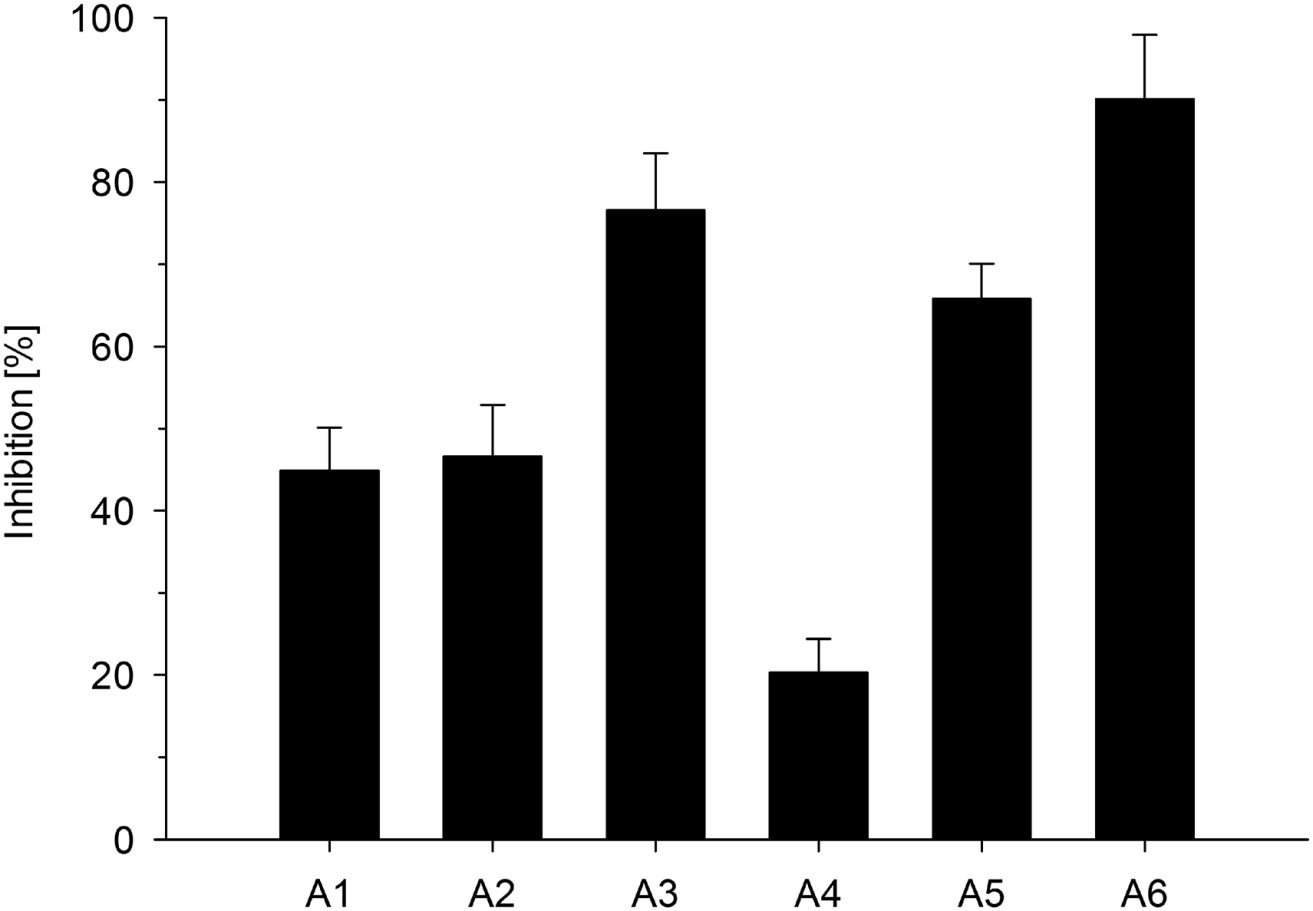
Treatment of *C*. *pneumoniae* elementary bodies with derivatives A1—A6. EBs were incubated with derivatives **A1**—**A6** at concentrations of 5 μM for 1 h. Suppression of infectivity of the EBs was determined after inoculation of HL cells and 72 h of incubation. (Results display the mean ± SEM).

A hallmark of AMPs and AMP derived compounds is their ability to act on metabolically quiescent bacteria by a membrane disruptive mechanism of action. We studied the impact of the β^2,2^-amino acid derivatives on the metabolically quiescent infectious EBs of *C*. *pneumoniae* that are able to resist both harsh environmental factors and treatment with antibiotics [32]. Infectious EB suspensions were treated with each of the derivatives **A1—A6** at 5 μM for 1 h prior to inoculation on HL cell monolayers in order to gain insight into susceptibility of the EB membranes to all derivatives. As shown in Fig 4, all six derivatives decreased *C*. *pneumoniae* EB infectivity, with **A3** and **A6** being the most potent derivatives in this respect, causing 77% and 90% inhibition. Treatment of EBs with **A4** and **A5** resulted in 20% and 66% reduction of infectivity. The two derivatives **A1** and **A2**, capable of suppressing *C*. *pneumoniae* inclusion counts in the context of cellular infections (see above) caused up to 50% decrease in infectivity upon EB pretreatment, indicating that these compounds, too, were able to target *C*. *pneumoniae* EB membranes. No HL cell detachment or morphological changes were observed during these experiments, indicating that the fractions of compounds to which HL cells were exposed in these conditions did not cause cytotoxic effects.

### Transmission electron microscopy (TEM)

**Fig 5:**
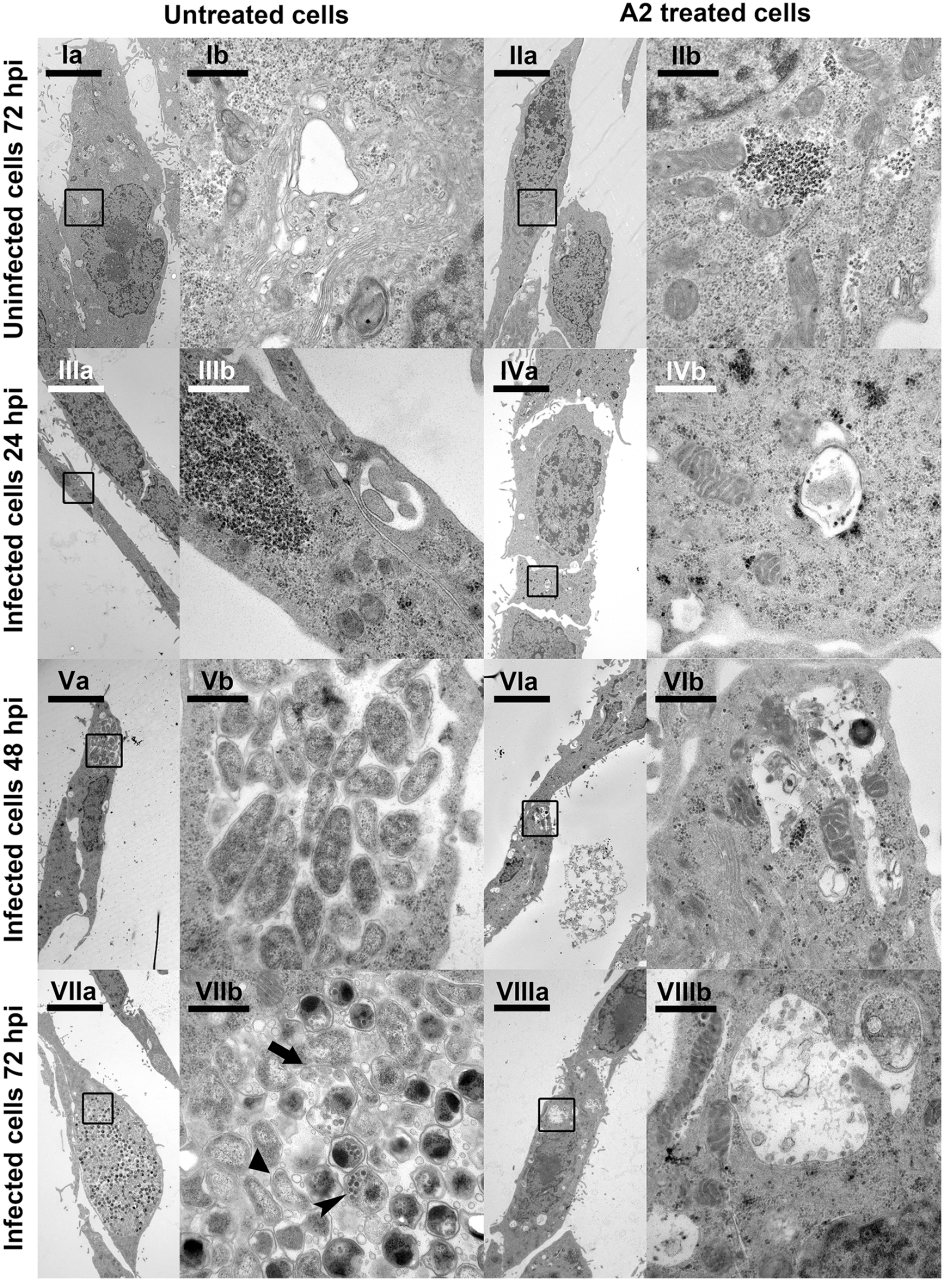
Transmission electron microscopy images of HL-cells. Images of untreated HL cells (uninfected and infected with *C*. *pneumoniae*) and HL cells treated with 2.5 μM of **A2** (uninfected and infected with *C*. *pneumoniae*) at different time intervals (hpi: hours post infection) were acquired (see Materials and methods for details). Scale bar in **a** series indicates 5 μm, whereas **b** series shows magnified regions with scale bar representing 0.5 μm. In **VIIb**: Triangle = Reticulate body, arrow = redifferentiating reticulate body, arrowhead = elementary body with miniature bodies.

TEM was used to trace intracellular changes upon *C*. *pneumoniae* infection and treatment with the lead derivative **A2** (Fig 5). Treatment of HL cells with 2.5 μM of **A2** over a time-span of 72 hpi did not result in remarkable changes in cell or organelle morphology compared with uninfected and untreated control cells (Fig 5Ia, 5lb, 5IIa and 5IIb). After infection with EBs and subsequent incubation for 24 h, RBs were visible in untreated control cells (Fig 5IIIa and 5IIIb) whereas affected inclusion vacuoles were visible in cells treated with **A2** (Fig 5IVa and 5IVb). By extending the incubation time to 48 h we observed an increased number of RBs per inclusion vacuole and expansion of the latter (Fig 5Va and 5Vb). Treatment with **A2** resulted in smaller inclusions, which only contained fragments of RBs and EBs (Fig 5VIa and 5VIb). After a 72 hpi course of the study, extended accumulations of RBs, redifferentiating RBs, and spherical EBs were visible in the untreated HL-cells (Fig 5VIIa and 5VIIb). Miniature bodies characteristic for *C*. *pneumoniae* were also visible. In contrast, no RBs or EBs were present in the **A2** treated cells, but inclusion vacuoles containing remnants of RBs and EBs after *C*. *pneumoniae* infection were visible (Fig 5VIIIa and 5VIIIb).

## Discussion

Despite some recent advances in diagnostics and basic biological studies, *C*. *pneumoniae* remains a challenging target for pharmacological intervention as illustrated by the high ratio of treatment failures with currently available antibiotics [6]. In contrast to most other respiratory pathogens, no resistant mutants of *C*. *pneumoniae* have been isolated from clinical samples to date. Even strains originating from patients with treatment failure do not show altered *in vitro* susceptibility profiles to antibiotics. Furthermore, previous *in vitro* studies do not show remarkable differences in antibiotic susceptibility between different *C*. *pneumoniae* reference strains or clinical isolates [33–35]. These observations indicate that persistence, rather than resistance, is responsible for the treatment challenges and emphasizes the need for novel types of antichlamydial agents not relying on bacterial replication.

In our earlier studies, various β^2,2^-amino acid derivatives have been shown to be active against multi-resistant bacteria and to be able to permeate through phospholipid membranes [22]. Furthermore, we have reported that biofilms formed by *Staphylococcus aureus* and *Escherichia coli* are susceptible to treatment with these β^2,2^-amino acid derivatives [36]. We have also demonstrated that some have anticancer activity by either attacking mitochondria in cancer cells or the cancer cell membrane and that this is related to differences in molecular structure [30]. The antimicrobial and permeability properties of β^2,2^-amino acid derivatives encouraged us to investigate if the intracellular pathogen *C*. *pneumoniae* is susceptible to these AMP derived compounds. Therefore, infected human epithelial HL cells as well as *C*. *pneumoniae* EBs were used in the screening.

A direct interaction of β^2,2^-amino acid derivatives with negatively charged heparan-sulfate and LOS containing bacterial membranes of EBs during the initial phase of infection can, to a certain extent, explain the anti-chlamydial effect of the derivatives. **A3** and **A6** were the most potent derivatives when applied to EBs prior to infection. Previous studies have shown that these two derivatives display pronounced membranolytic properties compared to other related β^2,2^-amino acid derivatives [24]. In contrast, **A1** and **A2** were non-toxic and both derivatives decreased *C*. *pneumoniae* infectivity when the bacterial EBs were treated prior to infection. Thus, interactions of **A1** and **A2** with *C*. *pneumoniae* EBs during the late attachment phase to HL cells (~ 2 hpi, onset of incubation) appear plausible. As a consequence, cell entry and development of infection was hampered.

**Fig 6:**
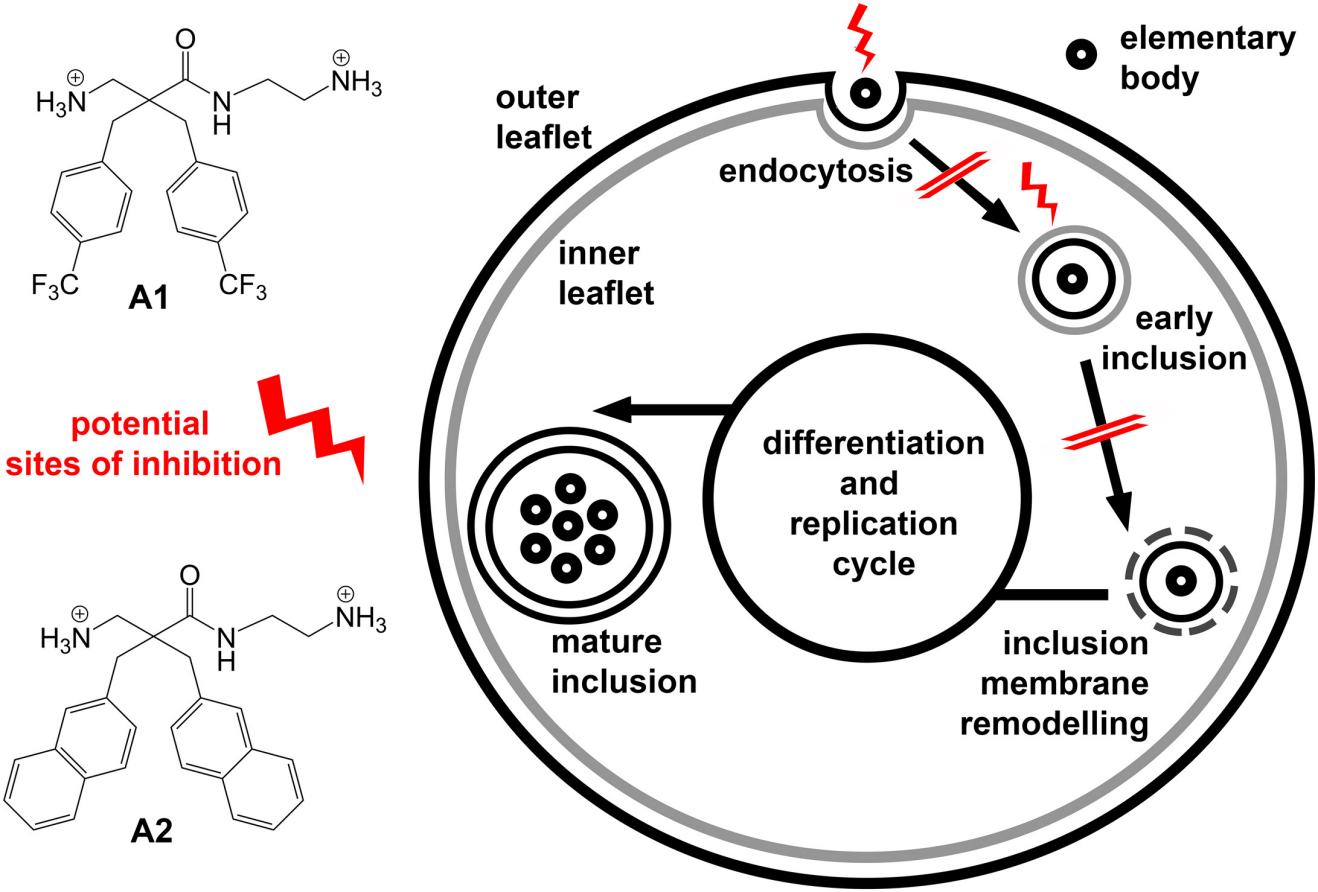
Schematic drawing of HL cell infection by *C*. *pneumoniae* and potential intervention points of β^2,2^-amino acid derivatives. The cytosol is surrounded by the inner (grey) and outer (black) leaflet of the cell membrane. The possible sites of inhibition of β^2,2^-amino acid derivatives during the *C*. *pneumoniae* infection cycle are indicated by lightning symbols. Inhibition of infection progress is indicated by double strikethrough.

Compared to other types of nonconventional anti-chlamydial compounds previously identified by us, such as flavonoids and betulin-derived compounds [27, 37, 38], the present β^2,2^-amino acid derivatives show similar or higher potencies against *C*. *pneumoniae*. Of note, the ability of flavonoles like quercetin, rhamnetin and morin to penetrate membranes seems crucial for their anti-chlamydial properties [27]. We thus hypothesize that the anti-chlamydial properties of **A1** and **A2** are also based on their ability to passively diffuse across the HL cell phospholipid bilayer and a subsequently form electrostatic interactions with *C*. *pneumoniae* bacterial and/or cellular inclusion membranes inside the infected HL-cells. We suspect that negative charges are present on endocytic vesicles containing *C*. *pneumoniae* EBs as reported for the inclusion vacuoles of a related pathogen *Chlamydia trachomatis* [39]. The negative charges on the endocytic vesicle originate from the membrane lipid phosphatidylserine, which is preferentially found on the inner leaflet of cell membranes. However, during endocytosis topological inversion of the phospholipid bilayer occurs and phosphatidylserine is located on the endosome exterior, as illustrated in Fig 6. According to the proposed mechanisms of actions for AMPs, the following event is interaction of the lipophilic side chains of the amphipathic β^2,2^-amino acid derivatives with the inclusion vacuole membrane and, upon disintegration, elimination of this protective niche needed for *C*. *pneumoniae* replication. We further hypothesize that the inclusion vacuole membrane and physiological processes connected to it are more susceptible to derivate interaction than the inner leaflet of the cell membrane, which is supported by our viability data.

The proposed mechanism of action is further supported by data acquired during the delayed administration assay. Time experiments showed that postponing the administration of **A1** or **A2** by only four hours (from 2 to 6 h) reduced drastically the anti-chlamydial effect (Fig 3A). This indicated that these derivatives suppress *C*. *pneumoniae* growth by affecting the events occurring in the early stages of the bacterial replication cycle. Similar observations have been made for the 21 amino acid long cell penetrating peptide Pep-1 and the 69 residue long intracellular expressed AMP CIT 1a [40, 41]. Both peptides were screened against *C*. *trachomatis* and displayed their highest activity during the initial stages of infection. The hours following the entry of *Chlamydia* into host cells are known to involve various remodeling processes of cellular membranes or organelles induced by the bacterium, as reviewed in Ronzone *et al*. [15]. The endocytic vesicle in which the EB is internalized is turned into an inclusion vacuole by incorporation of a unique combination of bacterial and host cell membrane proteins. Exocytic vesicles, multivesicular bodies and lipid droplets are trafficked into the inclusion vacuole to provide lipids as energy source and building blocks for the bacterium. Moreover, *Chlamydia* induced pathways prevent the fusion of the bacterium-endosomes with acidifying membrane organelles, which provides a neutral pH in the inclusion vacuole and protects the bacterium from degradation pathways [42].

Yeung *et al*. have reported that the related bacterium *C*. *trachomatis* actively manipulates the composition of endosome membrane charge and phospholipid composition [39]. While the abundance of phosphatidylserine in the outer leaflet of endosomal vesicles normally triggers the fusion of the vesicle with lysosomes, *C*. *trachomatis* is able to reorganize the vesicle by reducing the phosphatidylserine content and overall negative charge of the membrane’s outer leaflet. These changes were found to occur by 6 h post infection, which coincides with the observation from the delayed administration experiment of the current study (lack of inhibition when **A1** or **A2** were administered at 6 h or later). Of note, the β^2,2^-amino acid derivatives possess a considerably lower amount of positive charges (+2) compared to the intracellular expressed probes (+5) used by Yeung *et al*. [39]. This charge difference might be a possible explanation for the considerable drop in activity of **A1** and **A2** after 6 h. Thus, the impact of cationic charge on anti-chlamydial potency of future derivatives must be thoroughly evaluated since increasing the net positive charge might as well adversely affect the membrane penetrating properties of the β^2,2^-amino acid derivatives. These data further suggest a dual mechanism of action involving both destabilization of EBs and inclusion membranes. The results also indicate that **A1** and **A2** are active before *C*. *pneumoniae* enters its replication cycle, but further studies are needed to fully elucidate the detailed anti-chlamydial mechanism of action of these β^2,2^-amino acid derivatives.

## Conclusion

To our knowledge, the current work represents the first report on AMP-like compounds with activity against intracellular bacteria. The investigated β^2,2^-amino acid derivatives are chlamydicidal in nature and inhibit *C*. *pneumoniae* at clinical relevant concentrations. Thus, by exhibiting improved pharmacokinetic properties compared to many reported AMPs the β^2,2^-amino acid derivatives **A1** and **A2** demonstrate an important principle and are promising lead molecules for further drug development of agents with inhibitory activity against the intracellular pathogen *C*. *pneumoniae*.
